# 2-Chloro-*N*-(2,4-dichloro­phen­yl)­acetamide

**DOI:** 10.1107/S1600536809018753

**Published:** 2009-05-23

**Authors:** B. Thimme Gowda, Sabine Foro, Hiromitsu Terao, Hartmut Fuess

**Affiliations:** a Department of Chemistry, Mangalore University, Mangalagangotri 574 199, Mangalore, India; b Institute of Materials Science, Darmstadt University of Technology, Petersenstrasse 23, D-64287 Darmstadt, Germany; c Faculty of Integrated Arts and Sciences, Tokushima University, Minamijosanjima-cho, Tokushima 770-8502, Japan

## Abstract

The structure of the title compound, C_8_H_6_Cl_3_NO, contains two mol­ecules in the asymmetric unit. In each independent mol­ecule, the conformation of the N—H bond is almost *syn* to the *ortho*-chloro substituent and the conformation of the C=O bond is *anti* to the N—H bond. The mol­ecules in the crystal structure are linked into supra­molecular chains through N—H⋯O hydrogen bonding along the *a* axis.

## Related literature

For the preparation of the title compound, see: Shilpa & Gowda (2007 ▶); Pies *et al.* (1971 ▶). For related structures, see: Gowda, Foro & Fuess (2008 ▶); Gowda, Kožíšek *et al.* (2008 ▶); Gowda *et al.* (2009 ▶).
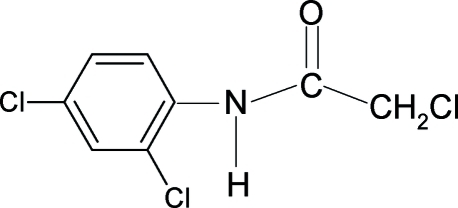


## Experimental

### 

#### Crystal data

C_8_H_6_Cl_3_NO
*M* *_r_* = 238.49
Monoclinic, 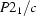

*a* = 4.7457 (5) Å
*b* = 12.9266 (9) Å
*c* = 31.879 (4) Å
β = 90.12 (1)°
*V* = 1955.6 (3) Å^3^
*Z* = 8
Mo *K*α radiation
μ = 0.89 mm^−1^
*T* = 299 K
0.48 × 0.05 × 0.05 mm

#### Data collection

Oxford Diffraction Xcalibur single-crystal diffractometer with a Sapphire CCD detector
Absorption correction: multi-scan (*CrysAlis RED*; Oxford Diffraction, 2007 ▶) *T*_min_ = 0.674, *T*_max_ = 0.957
7393 measured reflections
3590 independent reflections
1475 reflections with *I* > 2σ(*I*)
*R*_int_ = 0.077

#### Refinement

*R*[*F* ^2^ > 2σ(*F* ^2^)] = 0.080
*wR*(*F* ^2^) = 0.196
*S* = 0.91
3590 reflections
241 parameters
H atoms treated by a mixture of independent and constrained refinement
Δρ_max_ = 0.44 e Å^−3^
Δρ_min_ = −0.39 e Å^−3^

### 

Data collection: *CrysAlis CCD* (Oxford Diffraction, 2004 ▶); cell refinement: *CrysAlis RED* (Oxford Diffraction, 2007 ▶); data reduction: *CrysAlis RED*; program(s) used to solve structure: *SHELXS97* (Sheldrick, 2008 ▶); program(s) used to refine structure: *SHELXL97* (Sheldrick, 2008 ▶); molecular graphics: *PLATON* (Spek, 2009 ▶); software used to prepare material for publication: *SHELXL97*.

## Supplementary Material

Crystal structure: contains datablocks I, global. DOI: 10.1107/S1600536809018753/tk2452sup1.cif

Structure factors: contains datablocks I. DOI: 10.1107/S1600536809018753/tk2452Isup2.hkl

Additional supplementary materials:  crystallographic information; 3D view; checkCIF report

## Figures and Tables

**Table 1:** Hydrogen-bond geometry (Å, °)

*D*—H⋯*A*	*D*—H	H⋯*A*	*D*⋯*A*	*D*—H⋯*A*
N1—H1N⋯O1^i^	0.91 (7)	1.95 (7)	2.851 (7)	170 (6)
N2—H2N⋯O2^i^	0.77 (7)	2.11 (7)	2.872 (7)	168 (8)

Symmetry code: (i) 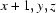.

**Fig. 1.**
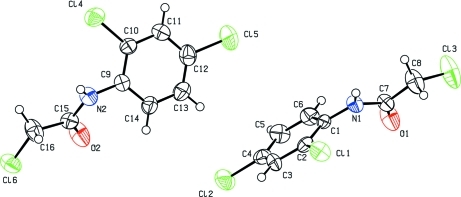
Molecular structures of the two independent molecules in (I), showing the atom labelling scheme. The displacement ellipsoids are drawn at the 50% probability level.

**Fig. 2.**
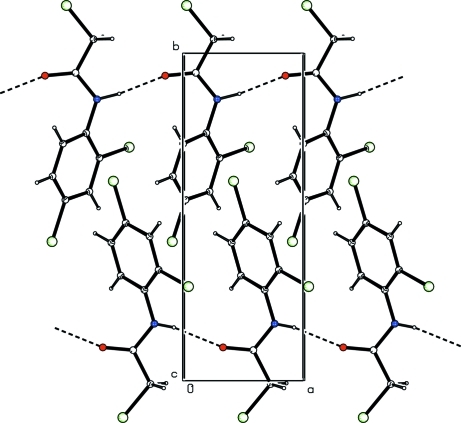
Molecular packing of (I) with hydrogen bonding shown as dashed lines.

## supplementary crystallographic information

### Comment

As part of a study into the effect of ring- and side-chain substitutions on the solid-state structures of aromatic amides (Gowda, Foro & Fuess, 2008; Gowda, Kožíšek *et al.*, 2008; Gowda *et al.*, 2009), in the present work the structure of the title compound (I) is described. There are two independent molecules in the asymmetric unit of (I), Fig. 1. The conformation of the N—H bond in each independent molecule is almost *syn* to the *ortho*-chloro substituent, similar to the *syn* conformation observed with respect to both the 2-chloro and 3-chloro substituents in 2-chloro-*N*-(2,3-dichlorophenyl)acetamide (Gowda *et al.*, 2008*a*). The conformation of the C=O bond is *anti* to the N—H bond, also similar to that observed in 2-chloro-*N*-(2,3-dichlorophenyl)acetamide. The N1–H1N···O1 and N2–H2N···O2 hydrogen bonding pack the molecules into supramolecular chains aligned along the a direction (Table 1, Fig. 2).

### Experimental

Compound (I) was prepared according to the literature method (Shilpa & Gowda, 2007). The purity of the compound was checked by determining its melting point. It was characterized by recording its infrared, NMR and NQR spectra (Shilpa & Gowda, 2007; Pies *et al.*, 1971). Single crystals of were grown by the slow evaporation of an ethanol solution of (I) held at room temperature.

### Refinement

The N-bound H atoms were located in difference map and their positional parameters were refined freely [N—H = 0.77 (7)–0.91 (7) Å]. The other H atoms were positioned with idealized geometry using a riding model [C—H = 0.93–0.97 Å]. All H atoms were refined with isotropic displacement parameters (set to 1.2 times of the *U*_eq_ of the parent atom).

To improve considerably the values of R1, wR2, and the GoF, eight reflections (-1 8 3, 0 10 4, 1 5 3, 2 5 0, 2 5 1, 2 5 3, 4 5 0, 1 1 28) were omitted from the final refinement.

### Crystal data

**Table:**

C_8_H_6_Cl_3_NO	*F*(000) = 960
*M**_r_* = 238.49	*D*_x_ = 1.620 Mg m^−^^3^
Monoclinic, *P*2_1_/*c*	Mo *K*α radiation, λ = 0.71073 Å
Hall symbol: -P 2ybc	Cell parameters from 1466 reflections
*a* = 4.7457 (5) Å	θ = 2.5–27.8°
*b* = 12.9266 (9) Å	µ = 0.89 mm^−^^1^
*c* = 31.879 (4) Å	*T* = 299 K
β = 90.12 (1)°	Needle, colourless
*V* = 1955.6 (3) Å^3^	0.48 × 0.05 × 0.05 mm
*Z* = 8	

### Data collection

**Table:**

Oxford Diffraction Xcalibur single-crystal diffractometer with a Sapphire CCD detector	3590 independent reflections
Radiation source: fine-focus sealed tube	1475 reflections with *I* > 2σ(*I*)
graphite	*R*_int_ = 0.077
Rotation method data acquisition using ω and φ scans	θ_max_ = 25.3°, θ_min_ = 2.5°
Absorption correction: multi-scan (*CrysAlis RED*; Oxford Diffraction, 2007)	*h* = −5→4
*T*_min_ = 0.674, *T*_max_ = 0.957	*k* = −15→11
7393 measured reflections	*l* = −38→38

### Refinement

**Table:**

Refinement on *F*^2^	Primary atom site location: structure-invariant direct methods
Least-squares matrix: full	Secondary atom site location: difference Fourier map
*R*[*F*^2^ > 2σ(*F*^2^)] = 0.080	Hydrogen site location: inferred from neighbouring sites
*wR*(*F*^2^) = 0.196	H atoms treated by a mixture of independent and constrained refinement
*S* = 0.91	*w* = 1/[σ^2^(*F*_o_^2^) + (0.0867*P*)^2^] where *P* = (*F*_o_^2^ + 2*F*_c_^2^)/3
3590 reflections	(Δ/σ)_max_ = 0.005
241 parameters	Δρ_max_ = 0.44 e Å^−^^3^
0 restraints	Δρ_min_ = −0.39 e Å^−^^3^

### Special details

**Table:**

Experimental. Absorption correction: CrysAlis RED (Oxford Diffraction, 2007) Empirical absorption correction using spherical harmonics, implemented in SCALE3 ABSPACK scaling algorithm.
Geometry. All e.s.d.'s (except the e.s.d. in the dihedral angle between two l.s. planes) are estimated using the full covariance matrix. The cell e.s.d.'s are taken into account individually in the estimation of e.s.d.'s in distances, angles and torsion angles; correlations between e.s.d.'s in cell parameters are only used when they are defined by crystal symmetry. An approximate (isotropic) treatment of cell e.s.d.'s is used for estimating e.s.d.'s involving l.s. planes.
Refinement. Refinement of *F*^2^ against ALL reflections. The weighted *R*-factor *wR* and goodness of fit *S* are based on *F*^2^, conventional *R*-factors *R* are based on *F*, with *F* set to zero for negative *F*^2^. The threshold expression of *F*^2^ > σ(*F*^2^) is used only for calculating *R*-factors(gt) *etc*. and is not relevant to the choice of reflections for refinement. *R*-factors based on *F*^2^ are statistically about twice as large as those based on *F*, and *R*- factors based on ALL data will be even larger.

### Fractional atomic coordinates and isotropic or equivalent isotropic displacement parameters (Å^2^)

**Table:**

	*x*	*y*	*z*	*U*_iso_*/*U*_eq_	
Cl1	0.5403 (4)	0.71319 (16)	0.00042 (6)	0.0507 (6)	
Cl2	−0.1002 (5)	0.42476 (17)	0.07378 (7)	0.0699 (8)	
Cl3	0.0189 (5)	1.14606 (18)	0.06668 (10)	0.0935 (9)	
O1	−0.1586 (10)	0.9317 (4)	0.06073 (19)	0.0672 (18)	
N1	0.2740 (11)	0.8593 (5)	0.06055 (18)	0.0362 (16)	
H1N	0.460 (14)	0.876 (5)	0.0583 (19)	0.043*	
C1	0.1840 (14)	0.7560 (5)	0.0644 (2)	0.0309 (17)	
C2	0.2935 (13)	0.6804 (6)	0.0379 (2)	0.0330 (18)	
C3	0.2098 (15)	0.5774 (6)	0.0412 (2)	0.0393 (19)	
H3	0.2862	0.5270	0.0238	0.047*	
C4	0.0105 (16)	0.5522 (6)	0.0710 (2)	0.047 (2)	
C5	−0.0950 (15)	0.6242 (7)	0.0982 (2)	0.046 (2)	
H5	−0.2243	0.6049	0.1186	0.055*	
C6	−0.0095 (15)	0.7241 (6)	0.0950 (2)	0.044 (2)	
H6	−0.0810	0.7727	0.1137	0.053*	
C7	0.0950 (14)	0.9405 (6)	0.0596 (2)	0.0386 (19)	
C8	0.2440 (16)	1.0429 (6)	0.0563 (3)	0.063 (3)	
H8A	0.3208	1.0505	0.0283	0.075*	
H8B	0.3997	1.0444	0.0761	0.075*	
Cl4	1.0368 (4)	0.28731 (17)	0.25087 (6)	0.0545 (6)	
Cl5	0.4118 (6)	0.60945 (19)	0.20155 (8)	0.0830 (8)	
Cl6	0.4903 (4)	−0.11251 (17)	0.16628 (7)	0.0586 (6)	
O2	0.3241 (10)	0.1017 (4)	0.1770 (2)	0.0701 (18)	
N2	0.7526 (12)	0.1738 (5)	0.1816 (2)	0.0422 (18)	
H2N	0.912 (15)	0.163 (6)	0.181 (2)	0.051*	
C9	0.6701 (14)	0.2773 (6)	0.1861 (2)	0.0335 (17)	
C10	0.7879 (14)	0.3385 (6)	0.2170 (2)	0.0381 (19)	
C11	0.7131 (15)	0.4406 (6)	0.2217 (2)	0.045 (2)	
H11	0.7958	0.4811	0.2425	0.054*	
C12	0.5141 (17)	0.4817 (6)	0.1952 (3)	0.049 (2)	
C13	0.3952 (15)	0.4215 (7)	0.1645 (3)	0.049 (2)	
H13	0.2595	0.4499	0.1469	0.059*	
C14	0.4723 (15)	0.3210 (6)	0.1595 (2)	0.044 (2)	
H14	0.3922	0.2817	0.1381	0.052*	
C15	0.5757 (15)	0.0933 (6)	0.1774 (2)	0.0374 (19)	
C16	0.7204 (15)	−0.0104 (6)	0.1735 (3)	0.062 (3)	
H16A	0.8307	−0.0229	0.1986	0.074*	
H16B	0.8496	−0.0079	0.1500	0.074*	

### Atomic displacement parameters (Å^2^)

**Table:**

	*U*^11^	*U*^22^	*U*^33^	*U*^12^	*U*^13^	*U*^23^
Cl1	0.0426 (12)	0.0541 (13)	0.0555 (13)	−0.0074 (10)	0.0135 (9)	−0.0047 (11)
Cl2	0.0955 (19)	0.0486 (15)	0.0657 (16)	−0.0266 (12)	−0.0015 (13)	0.0074 (12)
Cl3	0.0723 (18)	0.0432 (15)	0.165 (3)	0.0070 (13)	0.0398 (16)	0.0038 (16)
O1	0.018 (3)	0.043 (4)	0.140 (6)	0.002 (3)	0.005 (3)	−0.001 (3)
N1	0.016 (3)	0.034 (4)	0.058 (4)	−0.007 (3)	0.001 (3)	0.000 (3)
C1	0.029 (4)	0.030 (4)	0.034 (4)	0.006 (3)	−0.006 (3)	−0.002 (4)
C2	0.029 (4)	0.045 (5)	0.025 (4)	0.000 (3)	0.003 (3)	0.006 (4)
C3	0.040 (5)	0.028 (5)	0.050 (5)	0.002 (4)	0.003 (4)	−0.004 (4)
C4	0.049 (5)	0.051 (6)	0.040 (5)	−0.012 (4)	−0.012 (4)	0.004 (4)
C5	0.037 (5)	0.055 (6)	0.046 (5)	−0.015 (4)	0.013 (4)	0.002 (5)
C6	0.047 (5)	0.050 (6)	0.036 (5)	0.004 (4)	0.012 (4)	−0.007 (4)
C7	0.021 (4)	0.035 (5)	0.060 (5)	0.003 (4)	−0.001 (4)	−0.008 (4)
C8	0.033 (5)	0.043 (5)	0.113 (8)	−0.002 (4)	0.007 (4)	0.001 (5)
Cl4	0.0407 (12)	0.0626 (15)	0.0602 (13)	0.0013 (10)	−0.0087 (9)	−0.0003 (12)
Cl5	0.106 (2)	0.0461 (15)	0.097 (2)	0.0256 (14)	−0.0060 (15)	−0.0084 (14)
Cl6	0.0516 (13)	0.0489 (13)	0.0753 (16)	−0.0065 (11)	−0.0020 (11)	−0.0133 (12)
O2	0.020 (3)	0.047 (4)	0.143 (6)	0.010 (3)	−0.003 (3)	−0.013 (4)
N2	0.020 (3)	0.042 (4)	0.064 (4)	0.002 (3)	0.000 (3)	−0.003 (3)
C9	0.028 (4)	0.036 (5)	0.037 (4)	0.000 (3)	0.008 (3)	0.001 (4)
C10	0.031 (4)	0.043 (5)	0.041 (5)	−0.001 (4)	−0.001 (3)	0.001 (4)
C11	0.045 (5)	0.043 (5)	0.047 (5)	−0.003 (4)	0.000 (4)	−0.008 (4)
C12	0.054 (6)	0.044 (5)	0.051 (5)	0.012 (4)	0.011 (4)	0.001 (5)
C13	0.043 (5)	0.054 (6)	0.049 (5)	0.007 (4)	−0.007 (4)	0.006 (5)
C14	0.043 (5)	0.041 (5)	0.046 (5)	0.005 (4)	−0.004 (4)	0.004 (4)
C15	0.022 (4)	0.045 (5)	0.045 (5)	0.002 (4)	0.001 (3)	−0.005 (4)
C16	0.035 (5)	0.040 (5)	0.110 (8)	−0.004 (4)	−0.001 (5)	−0.003 (5)

### Geometric parameters (Å, °)

**Table:**

Cl1—C2	1.728 (7)	Cl4—C10	1.730 (7)
Cl2—C4	1.731 (8)	Cl5—C12	1.733 (8)
Cl3—C8	1.740 (8)	Cl6—C16	1.728 (8)
O1—C7	1.209 (7)	O2—C15	1.199 (7)
N1—C7	1.350 (9)	N2—C15	1.344 (9)
N1—C1	1.407 (9)	N2—C9	1.401 (9)
N1—H1N	0.91 (7)	N2—H2N	0.77 (7)
C1—C2	1.392 (9)	C9—C10	1.381 (9)
C1—C6	1.403 (9)	C9—C14	1.385 (9)
C2—C3	1.393 (10)	C10—C11	1.376 (10)
C3—C4	1.381 (10)	C11—C12	1.373 (10)
C3—H3	0.9300	C11—H11	0.9300
C4—C5	1.367 (11)	C12—C13	1.371 (10)
C5—C6	1.358 (10)	C13—C14	1.360 (11)
C5—H5	0.9300	C13—H13	0.9300
C6—H6	0.9300	C14—H14	0.9300
C7—C8	1.505 (11)	C15—C16	1.511 (10)
C8—H8A	0.9700	C16—H16A	0.9700
C8—H8B	0.9700	C16—H16B	0.9700
			
C7—N1—C1	123.3 (6)	C15—N2—C9	125.1 (6)
C7—N1—H1N	115 (4)	C15—N2—H2N	118 (6)
C1—N1—H1N	122 (4)	C9—N2—H2N	117 (6)
C2—C1—C6	117.4 (7)	C10—C9—C14	118.4 (7)
C2—C1—N1	119.9 (6)	C10—C9—N2	120.5 (6)
C6—C1—N1	122.6 (6)	C14—C9—N2	121.1 (6)
C1—C2—C3	121.2 (6)	C11—C10—C9	121.6 (7)
C1—C2—Cl1	120.1 (6)	C11—C10—Cl4	118.3 (6)
C3—C2—Cl1	118.7 (6)	C9—C10—Cl4	120.1 (6)
C4—C3—C2	118.2 (7)	C12—C11—C10	118.8 (7)
C4—C3—H3	120.9	C12—C11—H11	120.6
C2—C3—H3	120.9	C10—C11—H11	120.6
C5—C4—C3	121.8 (7)	C13—C12—C11	120.1 (7)
C5—C4—Cl2	120.3 (7)	C13—C12—Cl5	120.6 (6)
C3—C4—Cl2	117.9 (7)	C11—C12—Cl5	119.3 (7)
C6—C5—C4	119.4 (7)	C14—C13—C12	121.1 (7)
C6—C5—H5	120.3	C14—C13—H13	119.4
C4—C5—H5	120.3	C12—C13—H13	119.4
C5—C6—C1	121.8 (7)	C13—C14—C9	120.0 (7)
C5—C6—H6	119.1	C13—C14—H14	120.0
C1—C6—H6	119.1	C9—C14—H14	120.0
O1—C7—N1	123.5 (7)	O2—C15—N2	123.6 (7)
O1—C7—C8	123.5 (7)	O2—C15—C16	122.1 (7)
N1—C7—C8	112.9 (6)	N2—C15—C16	114.3 (6)
C7—C8—Cl3	111.9 (5)	C15—C16—Cl6	113.6 (5)
C7—C8—H8A	109.2	C15—C16—H16A	108.8
Cl3—C8—H8A	109.2	Cl6—C16—H16A	108.8
C7—C8—H8B	109.2	C15—C16—H16B	108.8
Cl3—C8—H8B	109.2	Cl6—C16—H16B	108.8
H8A—C8—H8B	107.9	H16A—C16—H16B	107.7
			
C7—N1—C1—C2	132.9 (7)	C15—N2—C9—C10	132.3 (8)
C7—N1—C1—C6	−48.9 (10)	C15—N2—C9—C14	−48.6 (10)
C6—C1—C2—C3	1.3 (9)	C14—C9—C10—C11	0.0 (10)
N1—C1—C2—C3	179.6 (6)	N2—C9—C10—C11	179.2 (7)
C6—C1—C2—Cl1	−178.2 (5)	C14—C9—C10—Cl4	−179.8 (5)
N1—C1—C2—Cl1	0.1 (8)	N2—C9—C10—Cl4	−0.7 (9)
C1—C2—C3—C4	1.2 (10)	C9—C10—C11—C12	0.7 (11)
Cl1—C2—C3—C4	−179.4 (5)	Cl4—C10—C11—C12	−179.5 (6)
C2—C3—C4—C5	−3.0 (11)	C10—C11—C12—C13	−0.5 (12)
C2—C3—C4—Cl2	178.1 (5)	C10—C11—C12—Cl5	178.6 (6)
C3—C4—C5—C6	2.3 (11)	C11—C12—C13—C14	−0.5 (12)
Cl2—C4—C5—C6	−178.9 (6)	Cl5—C12—C13—C14	−179.5 (6)
C4—C5—C6—C1	0.4 (11)	C12—C13—C14—C9	1.3 (12)
C2—C1—C6—C5	−2.1 (10)	C10—C9—C14—C13	−1.0 (11)
N1—C1—C6—C5	179.7 (7)	N2—C9—C14—C13	179.9 (7)
C1—N1—C7—O1	−2.1 (12)	C9—N2—C15—O2	0.2 (12)
C1—N1—C7—C8	178.6 (6)	C9—N2—C15—C16	−179.5 (7)
O1—C7—C8—Cl3	14.0 (11)	O2—C15—C16—Cl6	2.5 (11)
N1—C7—C8—Cl3	−166.8 (5)	N2—C15—C16—Cl6	−177.8 (6)

### Hydrogen-bond geometry (Å, °)

**Table:**

*D*—H···*A*	*D*—H	H···*A*	*D*···*A*	*D*—H···*A*
N1—H1N···O1^i^	0.91 (7)	1.95 (7)	2.851 (7)	170 (6)
N2—H2N···O2^i^	0.77 (7)	2.11 (7)	2.872 (7)	168 (8)

Symmetry codes: (i) *x*+1, *y*, *z*.
